# Sociodemographic and clinical features predictive of SARS-CoV-2 test positivity across healthcare visit-types

**DOI:** 10.1371/journal.pone.0258339

**Published:** 2021-10-14

**Authors:** Jimmy Phuong, Stephanie L. Hyland, Stephen J. Mooney, Dustin R. Long, Kenji Takeda, Monica S. Vavilala, Kenton O’Hara

**Affiliations:** 1 UW Medicine Research IT, University of Washington, Seattle, WA, United States of America; 2 Microsoft Research Cambridge, Cambridge, United Kingdom; 3 Department of Epidemiology, University of Washington, Seattle, WA, United States of America; 4 Department of Anesthesiology and Pain Medicine, University of Washington, Seattle, WA, United States of America; 5 Department of Pediatrics, University of Washington, Seattle, WA, United States of America; New Mexico State University, UNITED STATES

## Abstract

**Background:**

Despite increased testing efforts and the deployment of vaccines, COVID-19 cases and death toll continue to rise at record rates. Health systems routinely collect clinical and non-clinical information in electronic health records (EHR), yet little is known about how the minimal or intermediate spectra of EHR data can be leveraged to characterize patient SARS-CoV-2 pretest probability in support of interventional strategies.

**Methods and findings:**

We modeled patient pretest probability for SARS-CoV-2 test positivity and determined which features were contributing to the prediction and relative to patients triaged in inpatient, outpatient, and telehealth/drive-up visit-types. Data from the University of Washington (UW) Medicine Health System, which excluded UW Medicine care providers, included patients predominately residing in the Seattle Puget Sound area, were used to develop a gradient-boosting decision tree (GBDT) model. Patients were included if they had at least one visit prior to initial SARS-CoV-2 RT-PCR testing between January 01, 2020 through August 7, 2020. Model performance assessments used area-under-the-receiver-operating-characteristic (AUROC) and area-under-the-precision-recall (AUPR) curves. Feature performance assessments used SHapley Additive exPlanations (SHAP) values. The generalized pretest probability model using all available features achieved high overall discriminative performance (AUROC, 0.82). Performance among inpatients (AUROC, 0.86) was higher than telehealth/drive-up testing (AUROC, 0.81) or outpatient testing (AUROC, 0.76). The two-week test positivity rate in patient ZIP code was the most informative feature towards test positivity across visit-types. Geographic and sociodemographic factors were more important predictors of SARS-CoV-2 positivity than individual clinical characteristics.

**Conclusions:**

Recent geographic and sociodemographic factors, routinely collected in EHR though not routinely considered in clinical care, are the strongest predictors of initial SARS-CoV-2 test result. These findings were consistent across visit types, informing our understanding of individual SARS-CoV-2 risk factors with implications for deployment of testing, outreach, and population-level prevention efforts.

## Introduction

As of January 20, 2021, over 96 million confirmed cases of COVID-19 and over 2 million COVID-19-related deaths have been reported worldwide [1]. Despite Public health mandates, daily cases and deaths in the United States continue to climb at record-setting rates towards the predicted trajectory of COVID-19 transmission [2]. With the deployment of vaccines, national strategies have adopted ethical principles to prioritize those most vulnerable to COVID-19 effects [3]. Health systems contain a wealth of medical knowledge about their patient populations with expanded testing capacities for SARS-CoV-2 occurring in hospitals, outpatient clinics, and community drive-up testing sites [4]. Despite that, identifying features that explain transmission patterns and likelihood to test positive remains a technical barrier to optimal community outreach. For meaningful interventions against further COVID-19 spread and effects, vaccination efforts need to be informed with geographic and population characteristics learned from testing efforts to maximize benefits and minimize harm [3].

A review of published diagnostic and prognostic models for COVID-19 revealed substantial gaps in knowledge about factors contributing to individual outcomes [5]. In the early months of 2020, health systems allocated tests to people on the basis of symptomatic presentation or likelihood of exposure event, which introduced deficiencies in representing asymptomatic patients [6–9]. These studies often utilized clinical features attributable to the emergency and intensive care unit inpatients setting, but are seldom available in other healthcare settings [10–13]. Epidemiological information, insights about vulnerable population locations, and social determinants of health have since been highlighted as critical characteristics for equitable testing, vaccination, and phased-reopening strategies [3, 14–16]. Few studies to date examined patient-level social determinants of health (e.g., patient race/ethnicity, occupation category, recent employment status) and ecological factors (e.g., population density, median annual household income in patient ZIP code, or recent positivity rate in ZIP code at the time of test) in the Seattle Puget Sound region [17, 18]. No studies to date examined these features across healthcare visit-types along with clinical vital signs, medical and medication history for their contributing value to predict SARS-CoV-2 test positivity.

While seemingly obvious, geographic features of recent COVID-19 cases are not well-studied with regards to the spectrum of patient-level clinical features. Spatial analysis of COVID-19 incidence often used county-level or state-level aggregate or deidentified datasets to minimize reidentification risks [14, 19]. In general, patient clinical and spatial-temporal characteristics have not been accessible at finer resolutions. In response to COVID-19 research needs, nationwide initiatives like the National COVID-19 Cohort Collaborative (N3C) formed to create research-ready HIPAA Limited Datasets and enclave research infrastructure to facilitate reproducible modeling [20]. With this information now co-located together, critical next steps would be the analytical codebase and capacities to examine contributions of clinical and non-clinical features towards prediction of test positivity and further preventative efforts.

To better understand the value of routinely-collected EHR data, we examined the contribution of clinical, sociodemographic, and geographic features towards individualized risk of COVID-19 infection.

## Methods

### Study overview

We developed a retrospective cohort to assess the relative contributions of patient demographics, medical history information, and spatial factors to predict initial SARS-CoV-2 test results. We stratified the analysis based on the health system visit type (i.e. inpatient, outpatient, telehealth/drive-up testing site) and characterized model performance using all features as well as limited subsets of features. The University of Washington Institutional Review Board approved this study as minimal risk and waived consent requirements. All analyses were performed in a HIPAA-compliant compute environment. Our approach used an Observational Medical Outcomes Partnership (OMOP) structured dataset [21] for transferrable modeling and research with N3C efforts [20]. The codebase is available to facilitate further pattern identification with COVID-19 testing resources.

### Data collection

**Fig 1:**
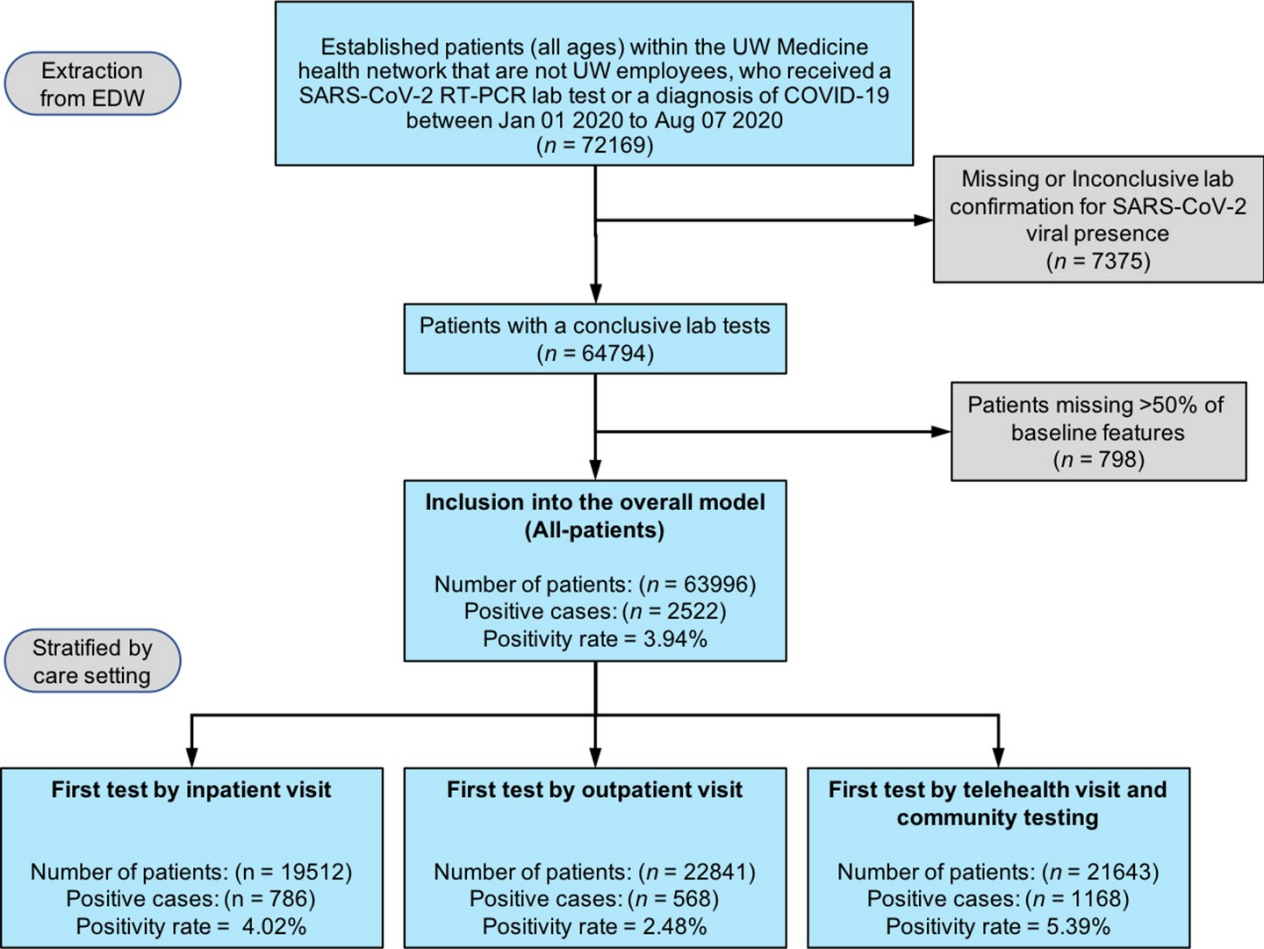
Patient inclusion and exclusion from model development and analysis. EDW = UW Medicine Enterprise Data Warehouse; Established patients = has one or more prior visit to the first SARS-CoV-2 test; SARS-CoV-2 = Severe acute respiratory syndrome coronavirus 2; COVID-19 = previously known as novel coronavirus disease 2019; RT-PCR = Reverse-transcriptase polymerase chain reaction assay; conclusive lab tests = results of ’positive’, ’detected’, ’negative’, or ’not detected’; Baseline features = ’last temperature measurement’, ’age’, ’race’, ’gender’, ’ethnicity’, ’occupation status’, ‘ZIP’, ’county’, and ’visit type’.

The CONSORT diagram depicting study inclusion/exclusion criteria is shown in Fig 1. In brief, UW Medicine patients who had at least one visit prior to initial SARS-CoV-2 RT-PCR testing between January 01, 2020 and August 7, 2020 were included. Exclusion of employee data was required for UW IRB approval. UW Medicine EHR data were abstracted into the OMOP common data model v5.3.1 for data analysis. Visit dates and times and ZIP code information were retained, and all other identifiers were removed.

### Data preparation

Patients were classified as test-positive or test-negative based on the lab-confirmed outcome of their first recorded SARS-CoV-2 RT-PCR test. Those with inconclusive results or missing essential features were omitted. We converted clinical EHR data into continuous, categorical, or binary-valued features and merged rare categories. For example, patient occupation information was categorized to 2018 Census Occupation Codes groups [22]. Highly collinear features (Pearson R > 0.8) were removed. Data collected after the time of first SARS-CoV-2 test collection were censored. For time-varying features such as clinical vital signs, transient symptoms, or laboratory test results, we included the most recent value and the mean from the two weeks prior to the test. Table A3 in S1 Appendix displays delays between most recent values and SARS-CoV-2 test. When test orders were not linked to a specific clinical encounter, we inferred the latest visit prior to the SARS-CoV-2 test was the clinical interaction resulting in test referral. Since not all telehealth interactions resulted in records in the visit database, we considered encounters where no visit was recorded up to 36 hours prior to the test to be telehealth visits.

We identified patient residential ZIP code from billing records and linked them to the American Community Survey’s 2018 ZIP Code Tabulation Area (ZCTA) to identify population density (person per sq. mile) and median household income. For each patient, as a proxy for disease prevalence near the patient’s home, we computed the positivity rate of all test results from the same ZIP code during the preceding two weeks. For the first test from each ZIP code, we treated this feature as missing.

Ultimately, 186 features were available for analysis. Further details on the processing steps are provided in the S1 Appendix.

### Modeling and analyses

We developed a binary classifier to predict the outcome of a patient’s initial SARS-CoV-2 RT-PCR test. To develop this model, we split data into three fixed partitions: training, validation, and testing (referred to as the “evaluation set”). we first withheld a random 20% of the dataset for final model performance assessment (the ‘evaluation set’). From the remaining 80%, we randomly subsampled data in a 4:1 fashion into training and validation subsets, respectively.

Our primary models were GBDT implemented using the LightGBM library [23]. GBDT have demonstrated strong performance in clinical prediction tasks, including pretest probability of SARS-CoV-2 positivity [10–12]. More details, including hyperparameters of the GBDT, are provided in the S1 Appendix.

Our primary analysis characterized overall model performance across all visit types. Primary metrics for model performance were AUROC curves, which compare model sensitivity versus specificity, and AUPR curves, which compare positive predictive value (PPV) to sensitivity. These complementary metrics offer insight into the expected false positive rate of the model in contexts where positive cases are rare. For all model evaluations, we report mean AUROC and 95% confidence intervals computed using 1000 bootstrap resamples of the evaluation set. Imputation of missing data was not performed as GBDT natively handles missingness as potentially informative, appropriate for a clinical setting in which the choice to order a test reflects clinical judgment.

To characterize model performance across clinical contexts, we stratified patients by visit type (inpatient, outpatient, and telehealth). To quantify the extent to which a given feature contributed positively or negatively to model prediction in these clinical settings, we computed SHAP values [24]. Using a mean absolute SHAP value, we indicate the average magnitude of impact of that feature on the model’s predictions. This flexible framework allowed us to quantify feature importance even for non-linear models, such as GBDT.

Finally, we examined the likely impact of data accessibility on model use in these settings by training variants using subsets of features representative of those expected to be available in inpatient, outpatient, or community testing scenarios. The intention of these feature sets was to represent the information likely to be accessible for real-word application of the model in that clinical environment. To do this, we first grouped the features into categories (“Chronic conditions”, “Geography”, “Drugs”, “Demographics”, “Insurance”, “Labs”, “Symptoms”, and “Vitals”), and then associated these categories with one of three feature sets: 1) *minimal* (those expected to be available in all settings including community drive-up environments, including demographic information, signs and symptoms, recent temperature checks, public and case statistics based on the patients’ geography), 2) *intermediate* (those available in a typical outpatient setting, additionally including chronic medical conditions, prescription medications, vital signs, and insurance/payment information), or 3) *full* (complete information as available in an inpatient setting, including provider order entry data about the indication for testing). Table A1 in S1 Appendix contains the full mapping of individual features to these feature sets.

To validate our choice of GBDT as a primary modeling strategy, we performed comparisons with logistic regression using mean imputation to account for missing features (Fig A1 in S1 Appendix). Python code used for the data preprocessing and analysis is available from: https://github.com/microsoft/sars-cov2-pretest-probability.

## Results

### Study population

Ultimately, records for 63 996 patients were available for analysis. Similar numbers of included patients were referred for testing from inpatient, outpatient, and telehealth visits. Data preprocessing is summarized in S1 Appendix. Fifty one percent were female and 37% (*n* = 33747) were non-White (Table 1). Approximately four percent *(n* = 2522) tested positive for SARS-CoV-2 at the time of first testing, with observed differences by race, ethnicity, occupation status, and geography.

**Table 1:** Key modeling variables and their availability in the study population.

Key variables	Missingness, n (%) of 63 996	Study population, n (%) of 63 996	Mean (sd) of study population	Positive cases, n (%) of 2522	Mean (sd) of cases
**Demographics**					
Age (years)	0 (0)		47 (19)		45 (20)
Sex = female	0 (0)	32826 (51)		1224 (49)	
Ethnicity = Hispanic	10763 (17)	5298 (9.9)		582 (29)	
Race					
White		40249 (63)		1248 (49)	
Black or African American		5896 (9)		345 (14)	
Asian		5659 (8.8)		216 (9)	
American Indian | Alaska Native		1007 (1.6)		79 (3)	
Native Hawaiian | Pacific Islander		735 (1.1)		38 (2)	
Unknown		10450 (16)		596 (24)	
Occupation status					
Full time		16438 (27)		521 (21)	
Not employed		14179 (22)		555 (22)	
Retired		9090 (14)		294 (12)	
Part time		2113 (3.3)		91 (4)	
Student (Full time)		1822 (2.8)		62 (3)	
Self Employed		1705 (2.7)		49 (2)	
On Active Military Duty		18 (0)		0 (0)	
Unknown		18589 (29)		946 (38)	
**Vitals and labs**					
Glucose (mg/dL) in s/p	27636 (43)		111 (39)		118 (47)
Albumin (g/dL) in s/p	33537 (52)		4.2 (0.4)		4.0 (0.5)
Leukocytes (10^3 count/μL) in blood	27295 (43)		8.2 (3.9)		7.4 (3.4)
Platelets (10^3 count/μL) in blood	27233 (43)		243 (86)		237 (86)
Days since respiratory rate measured	30839 (48)		8 (14)		10 (15)
Heart rate (bpm)	30228 (47)		79 (16)		81 (16)
DBP (mmHg)	30292 (47)		76 (13)		75 (13)
Weight (kg)	39653 (62)		81 (20)		80 (19)
Height (meters)	40272 (63)		1.7 (0.1)		1.67 (0.1)
**Geography**					
2-week test positivity in ZIP (%)	46 (0)		4.1 (3.7)		6.3 (4.9)
Median household income in ZIP ($)	2587 (4)		82187 (24538)		77383 (22196)
Population density (person/sq. mile)	2530 (4)		6493 (6366)		6105 (4954)
**Other variables**					
Visit type of first test = inpatient	0 (0)	19512 (30)		786 (31)	
Visit type of first test = outpatient	0 (0)	22841 (36)		568 (23)	
Visit type of first test = telehealth	0 (0)	21643 (34)		1168 (46)	
Insurance type = commercial	10825 (17)	24985 (39)		602 (24)	
Insurance type = military	10825 (17)	838 (1.3)		20 (1)	

2-week test positivity in ZIP = the percent of patients in ZIP code who test positive within the prior 2-weeks; DBP = Diastolic blood pressure; bpm = beats per minute; s/p = serum or plasma.

### Performance in predicting the first SARS-CoV-2 test

**Fig 2:**
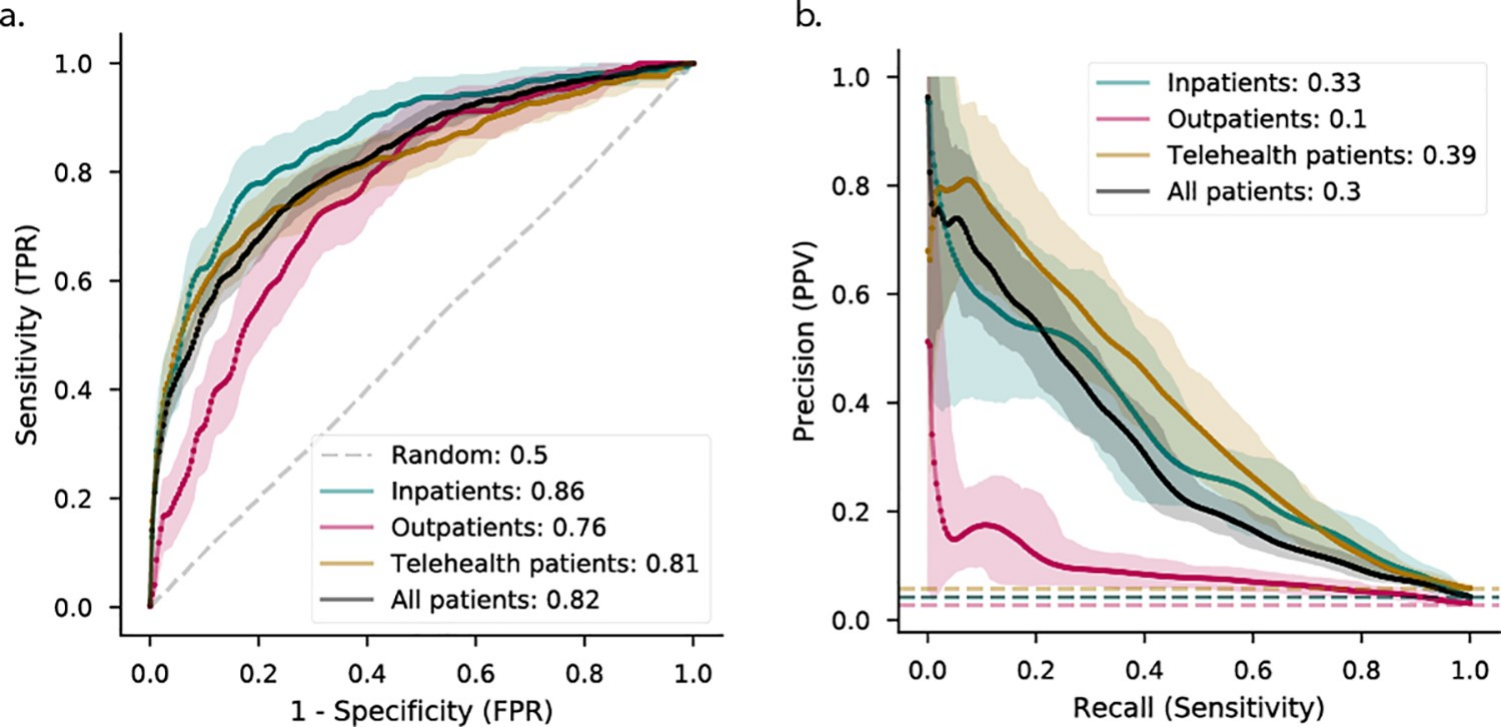
Receiver-operating characteristic (ROC) and precision-recall curves. (A) The model achieves generally high performance, which is unevenly distributed by visit type–performance in inpatient settings is the best. (B) Precision-recall curves indicate significant class imbalances, reflective of the approximate 2–4% positive rate, where the outpatient set observed the worst imbalance and fewest positive cases. Dashed lines indicate the reference performance of a random classifier. TPR = True positive rate; FPR = False positive rate; PPV = positive predictive value. Solid lines indicate the mean of 1000 bootstrap replicates of the evaluation set, while shaded areas are 95% confidence intervals.

The primary model, fitted across all patients, achieved high discriminatory performance (AUROC, 0.82 [95% CI, 0.80–0.84]) for prediction of positive versus negative first-test result (Fig 2A). This model performed better among inpatients (AUROC, 0.86 [CI, 0.83–0.89]) than among outpatients (AUROC, 0.76 [CI, 0.72–0.80]) and telehealth patients (AUROC, 0.81 [CI, 0.78–0.84]). Despite the low fraction of positives across visit types, precision-recall curves indicate that the model distinguishes true positive cases with higher-than-expected sensitivity and positive predictive value in inpatient and telehealth but not outpatient groups (Fig 2B). Telehealth held the highest positive predictive value among the visit types. The low positive predictive value among outpatients likely relates to the lower overall positive rate of tests performed in this group (2.4%).

### Identification of important features

**Fig 3:**
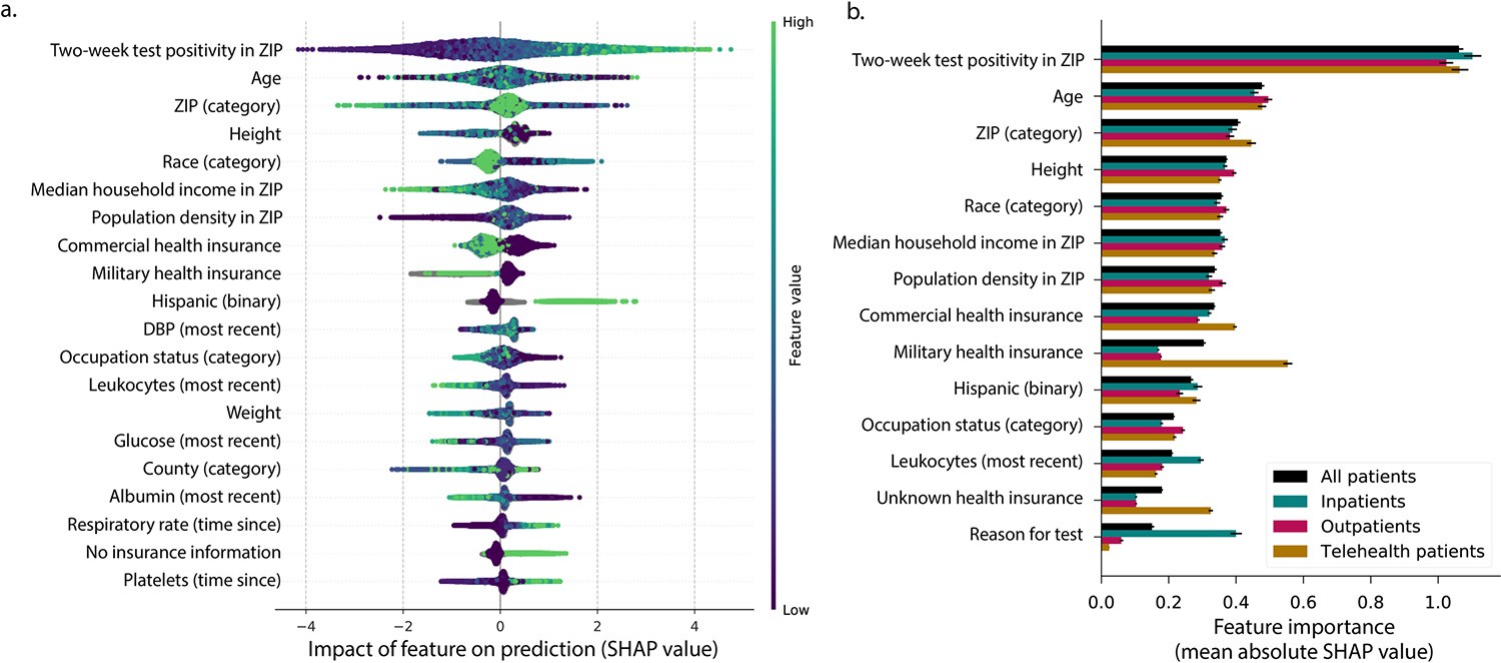
Feature-wise importance for predicting COVID-19 status in all patients and care-setting cohorts. (A) The top 20 individual features are rank-ordered by mean absolute SHAP value, the measure of proportional importance towards the prediction of positive test outcomes. Each point represents a single patient in the test set, colored by the value that feature took on in that patient. For continuous features, color indicates feature value of green (high) to purple (low). For categorical/binary features, color indicates different categories. Gray indicates missing value. The x-axis is the SHAP value; positive values increase the model prediction and vice-versa. (B) The aggregate feature importance is reported as mean absolute SHAP value. Importance is stratified by visit type; ranking is based on importance to “All patients” cohort. Some features have a cohort-specific importance and greater role in prediction. (most recent) = most recent record prior to the SARS-CoV-2 test; DBP = Diastolic blood pressure.

Fig 3A shows individual SHAP values for the top 20 most important features. Each point represents a single patient in the test set. Positive SHAP values indicate that the value of that feature (for that patient) prompted the model to assign a higher probability of positive SARS-CoV-2 test outcome.

The feature “two-week test positivity in ZIP” was the most informative, with high recent positivity rates increasing positive predictions. Although age was also informative, the relationship between age and predicted test positivity was not monotonic, as depicted by the mottled coloring along the x-axis. Fig A2 in S1 Appendix shows SHAP values for informative features within visit types.

Fig 3B reports the mean absolute SHAP value, stratified by visit type, showing a general consistency of feature importance across visit types, with notable exceptions. For example, although “reason for test” did not rank among the top features across all patients, Fig 3B demonstrates that it is informative for inpatients (in practice, ‘reason for test’ was typically available only for inpatients in this dataset). Similarly, leukocyte count appears informative when available.

### Role of feature categories and data availability in treatment settings

**Fig 4:**
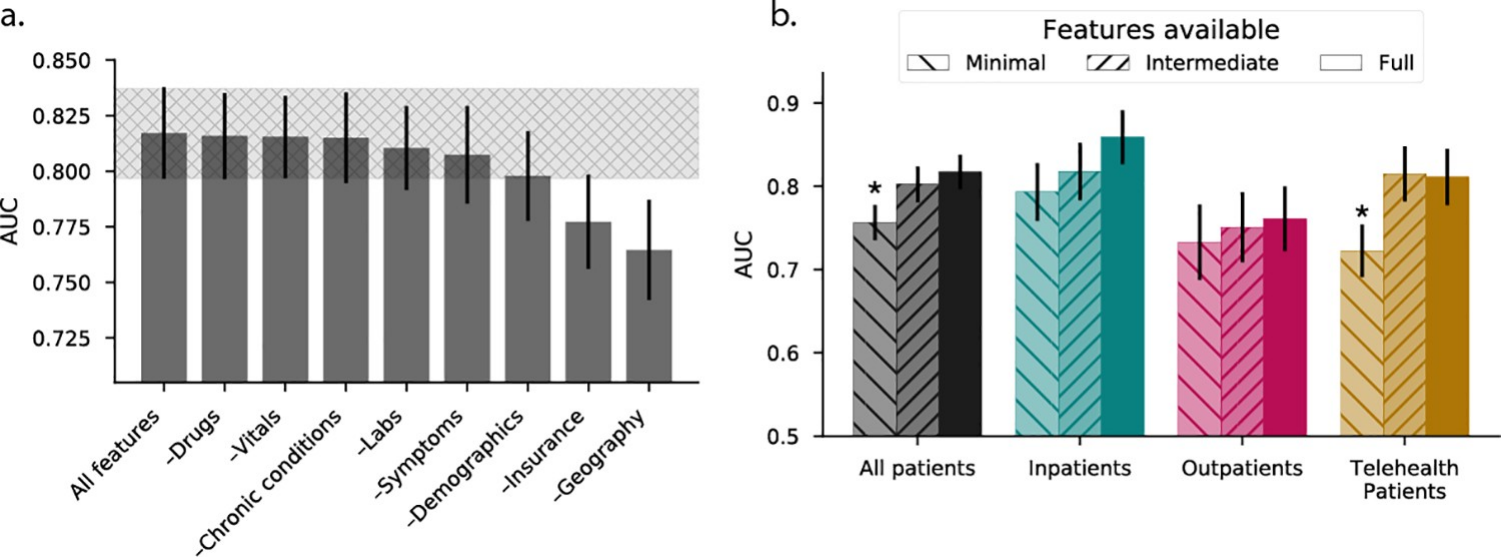
Group-wise feature importance for prediction of SARS-CoV-2 test outcome by visit types. (A) Across the all patient cohort, the feature categories were ablated to rank-order the group-wise importance towards the model performance. The hatched region indicates the 95% confidence interval using all features. (B) Performance of three hypothetical models using subsets of features (minimal, intermediate, full feature sets) shows differences in model performance when stratified by visit type (color, x-axis). AUC = area-under-the-receiver-operating-characteristic curve; *Minimal* feature set = symptoms, last available temperature, race, ethnicity, gender, age, occupation, zip, county, population density, two-week test positivity in zip, median household income in zip, type of visit. *intermediate* feature set = minimal features + chronic conditions, drugs, blood pressure, heart rate, respiratory rate, and oxygen saturation. *Full* feature set = intermediate features + “reason for test” and all laboratory test results excluding those in class B. The full feature set corresponds to all available features. Asterisk indicates the 95% confidence interval does not overlap with using the full feature set.

We grouped individual features into prespecified categories to examine the overall importance of these information classes on prediction. Ablation testing indicated that geography was the most informative category (Fig 4A), demonstrating the greatest degradation in performance when removed from the model, followed by insurance status and demographic factors. In contrast, selectively removing information regarding chronic medical conditions, vital sign measurements, and medications results did not significantly impact performance. Across visit types, use of more comprehensive feature sets uniformly improved the accuracy of predictions, although even the *minimal* set (representing data expected to be available for use in telehealth/community testing sites) maintained useful discriminatory power (AUROC > 0.7) (Fig 4B).

## Discussion

In this study, we demonstrate that routinely collected EHR data can be used to build predictive models of initial SARS-CoV-2 RT-PCR test results with high discriminative performance across visit types. Overall, while a combination of both sociodemographic and clinical features yielded the highest predictive performance, geographic, demographic, and socioeconomic features were more strongly associated with initial SARS-CoV-2 test positivity than clinical characteristics across all visit types examined. Recent test positivity rate in home ZIP code was the most informative single factor. We also found that models using only a minimal set of variables, commonly available in most test settings, retained useful discriminative power. These findings demonstrate that effective risk-stratification is possible in drive-up test settings and at the population level, where individual clinical data (laboratory values, past medical history) are not typically available.

Unlike prior studies [10–13], this study explores the role of social determinants of health, ecological factors along with the clinical features that are routinely available in EHR data across inpatient, outpatient, and telehealth visit types. The best performing prior model for prediction of SARS-CoV-2 positivity among ED patients used pre-pandemic controls and examined vital sign and blood gas measurements only among patients receiving blood tests and subsequently admitted to the hospital, achieving an AUROC of 0.94 [10]. This approach limits generalizability to patients who undergo testing to in-hospital settings. In contrast, our model, which included all visit types, produced comparable performance to a study using only blood gas measurements collected within 48 hours of the SARS-CoV-2 test [11] and markedly higher performance than models without such time-constraints on the recency of available clinical data (AUROC, 0.66) [12]. Present findings extend the applicability of prior works to a broader range of clinical scenarios and provide general insight into the factors contributing to COVID-19 transmission.

A unique aspect of this study was the derived feature “two-week test positivity in ZIP,” which emerged as the most important single feature across all visit types and contributed more to the prediction than clinical data. This feature is a useful proxy for recent disease prevalence in the area and its relative importance in this context is consistent with the known epidemiology of community-based transmission [25, 26]. We selected a 2-week period for incident rate calculation based on viral incubation rates and associated guidelines for containment and quarantine [16]. This construct provides a convenient and lightweight representation of up-to-date regional transmission dynamics that has the potential to easily transfer across other regions and times.

Other geographically derived factors also featured prominently within the list of important model features. Most notable was the feature “median household income in ZIP”, with lower income being associated with higher pretest probability of positivity. Alongside the presence of commercial health insurance and occupation status, these features point to the importance of including socioeconomic factors in models of test positivity. High population density areas have experienced high exposure risks and transmission levels, which may in part be due to high-density group occupancy [27, 28]. While it is difficult to draw strong conclusions regarding the factors underlying these observations, they are consistent with other studies that highlight effects of neighborhood-specific socioeconomic factors on COVID-19 prevalence [14, 19, 29, 30].

The relative importance of geographic features in our model is key because these features are typically available in telehealth/drive-up test settings. Our finding that model discrimination varied by treatment setting was unsurprising but is important. Data availability (e.g., presence of laboratory values), reasons for testing, and underlying health status vary greatly between inpatient, outpatient, and telehealth settings. Indeed, approximately half of the total cohort did not have a clinical history meriting antecedent vitals and laboratory studies. Researchers and practitioners considering implementation of such models should consider treatment setting carefully when evaluating their application and potential performance across these distinct clinical settings. Using this approach, we demonstrate that data routinely available to testing facilities, hospital systems, and public health officials can be usefully derived using readily available EHR data without recourse to other disparate sources [31]. Heightened risk may indicate need for targeted outreach to specific patient groups, deployment of mobile testing services to specific communities, and expedited laboratory processing tiers for individual samples [32]. Consistently low risk levels may be good candidates for pooled testing or environmental surveillance strategies [33–36].

While clinical measures, such as vitals and laboratory measures, were not the focus of this study, these measures did contribute measurably to the predictive performance of models in some settings and warrant examination. Several clinical markers predictive of SARS-CoV-2 positivity in this study have previously been associated with severe COVID-19, including decreased white blood cell count [10, 11, 37, 38], platelet count [10, 38–40], serum albumin levels [40–42], respiratory rate [43], and diastolic blood pressure [38, 43]. A strong predictive feature newly identified in this study was patient height, where shorter stature was associated with increased risk of SARS-CoV-2 positivity. It has been hypothesized that children and shorter adults may observe increased exposure risks due to prolonged droplet retention at lower breathing heights [44, 45]. Although patient height is related to body mass index, a known risk factor for severe infection [46], patient weight was separately accounted for in the model, suggesting that patient height is an important risk factor independent of body composition, age, and other demographics. As virulent strains continue to pose emergent threats, for those without immunity and overwhelming risk factors, clinical measures and blood gas findings may provide perspective on the patients’ health status and whether they are at greater propensity for severe COVID and hospitalization [10, 38]. Unfortunately, these clinical measures are not reliably available in outpatient and community testing settings.

In the United States, COVID-19 vaccination strategies have prioritized social determinants among their guiding principles [3]. These efforts and ongoing work to identify emergent SARS-CoV-2 variants of clinical importance will continue to necessitate current, regional information on populations at increased risk of SARS-CoV-2 transmission and development of severe manifestations of COVD-19 once acquired. Our model provides an efficient method of identifying those with a high probability of newly testing positive and represents a potential approach to characterizing vulnerable populations [19, 27, 47]. In practice, different healthcare settings have varying capacities for risk assessment tools. Our approach used an OMOP limited dataset structure, which bypasses the usual interoperability barriers of EHR-based research [20, 21], providing the data structure necessary for portability to other health systems.

This study has some limitations. Data analyzed were drawn from a clinical care database wherein patient referral for testing was influenced by regional guidelines that impact broader generalizability [48]. For example, in Washington State, older adults were prioritized for testing early in the pandemic and the reported role of age as a predictive factor may vary by time and region. Similarly, patients for whom English is not a first language have reported barriers to test access [17]. SARS-CoV-2 presence testing for UW Medicine employees were excluded from this study. Findings based on the catchment area of a single health system may not generalize because of ascertainment and selection bias. Our proxy for home environment, ZIP code, is imperfect. Patients who spent time in other ZIP codes and may have contracted disease there. Moreover, ZIP codes are socially and environmentally heterogenous and US Census ZCTA is an imperfect representation of the international Postal ZIP code [49, 50].

In conclusion, our study demonstrates that routinely collected non-clinical features in EHR contribute significantly to prediction of initial SARS-CoV-2 test positivity across a variety of visit types and clinical testing scenarios. The key role of sociodemographic features in the outcome of SARS-CoV-2 testing has implications not only for prediction of individual test positivity but also for objective deployment of testing, outreach, and population-level prevention efforts.

## Supporting information

S1 Appendix Description of data preprocessing, model diagnostics, and sensitivity analyses for prediction of COVID status. (DOCX)

S1 Checklist (DOCX)
