# Supplementary material for: Sociodemographic and clinical features predictive of SARS-CoV-2 test positivity across healthcare visit-types
Source: PLoS One. 2021 Oct 14;16(10):e0258339. doi: 10.1371/journal.pone.0258339 (PMC8516280; doi:10.1371/journal.pone.0258339)
Supplement: S1 Appendix — (DOCX) [file pone.0258339.s001.docx]

**S1 Appendix - Clinical and Sociodemographic Features Predictive of SARS-CoV-2 Test Positivity Across Healthcare Visit-types: A Retrospective Cohort Analysis**

Jimmy Phuong, MSPH, PhD*; Stephanie L. Hyland, PhD*; Stephen J Mooney, PhD; Dustin R Long, MD; Kenji Takeda, PhD; Monica S Vavilala, MD; Kenton O’Hara, PhD

**Supplementary materials and methods**

## Patient identification

## Data preprocessing

## Visit type mapping

## Patient-level filtering

## Category-specific variable processing

- Clinical features – lab test results and vitals
- Conditions – chronic, signs and symptoms
- Geography
- Drugs
- Demographics
- Insurance
- Test indication

## Imputation and feature-level filtering

## Modelling details

- Fig A1. Comparison of performance between gradient-boosted tree model and logistic regression.

## Implementation details

## Individual feature importance stratified by visit type

- Fig A2. SHAP values for top features based on the inpatient cohort only.
- Table A1. Table of features.
- Table A2. Minimum and maximum thresholds set for features.
- Table A3. Average delay between most recent lab measurements and date of SARS-CoV-2 test.

## References

## Patient identification

72,169 patients were identified as having at least one or more prior medical encounter with the UW Medicine Health system (UW Medical Center, Northwest Hospital, Harborview Medical Center, Neighborhood Clinics, and Seattle Cancer Care Alliance) and receiving a SARS-CoV-2 viral RNA presence RT-PCR test between January 1, 2020 and August 7, 2020. A HIPAA limited data set was generated using the Observational Medical Outcomes Partnership (OMOP) database schema v5.3.1^1^ from the UW Medicine Enterprise Data Warehouse for these patients. This data set contains the patient prior medical history for those who were screened or tested for COVID-19, but does not include patients who were UW Medicine employees or were tested for COVID-19 on behalf of another health system.

The dataset includes patient medical history as early as January 1, 2010, encompassing patient demographics, medical diagnosis, procedures, observational vital signs, medication history, and laboratory measurements. Variables for insurance / payment method, occupational category, reason for the SARS-CoV-2 test (test indication), patient ZIP code on-file, population density in the ZIP code, and mean annual household income in the ZIP code were included.

### Data preprocessing

Preprocessing generated a patient-level table with 186 features (Table A1) and 63996 unique patients, where each row was a patient that had received a SARS-CoV-2 RT-PCR test (OMOP concept id: 706163). Implausible values according to Table A2 were removed. The next sections provide further detail on the preprocessing steps.

## Visit type mapping

We enforced a restriction that SARS-CoV-2 tests must occur during or within 36-hrs of a visit. In cases where patients had a SARS-CoV-2 viral presence test performed without a documented encounter within 36-hrs, we assumed these measurement events as associated to telehealth-type visits. Outpatient visits are defined as patient encounters taking place at neighborhood clinics, urgent care and drop-in clinics, and rehabilitative therapy care visits. Inpatient visits are defined as overnight hospital visits, admissions to the emergency room, and admissions to the intensive care units. Telehealth visits include telehealth / telemedicine virtual appointments, phone calls with the patient, and/or virtual interactions resulting in subsequent medical actions, such as SARS-CoV-2 test ordered for collection at community-based and drive-through testing sites.

## Patient-level filtering

Patients with inconclusive SARS-CoV-2 test results were excluded. Patients missing more than 50% of the features 'last temperature measurement', 'age', 'race', 'gender', 'ethnicity', 'occupation status', 'ZIP', 'county', and 'visit type” were removed.

## Category-specific variable processing

We provide details on the processing of each category of features in the following:

##### *Clinical features – lab test results and vitals*

For clinical vital sign observations (i.e., pulse, blood oxygen levels, systolic and diastolic blood pressure, and temperature), we took the most recent measurement prior to the test, the time between the most recent measurement and the test, and the average value for the prior week to the test. Lab tests (e.g., blood glucose levels, hemoglobin count, platelet count, and blood chemistry panels) were treated similarly, but included the most recent value and average value from the two weeks prior to the test.

##### *Conditions – chronic, signs and symptoms*

We inferred chronic conditions, signs and symptoms based on diagnosis codes. If a code was not recorded, we assumed the condition was not present. For chronic conditions, we considered if a code had ever been recorded prior to the test. For signs and symptoms, the condition had to be recorded in the two weeks prior to the test.

##### *Geography*

Geographic features consist of the ZIP code and county, and ZIP-based features such as population density and median household income. We mapped rare ZIP codes and counties (containing less than 1% of the patients) to “other”. To obtain zip-level metadata we cross-referenced the American Community Surveys 2018 ZIP Code Tabulation Area (ZCTA) dataset. Our derived feature of “2-week test positivity in ZIP” was estimated empirically for each patient from the dataset. If a patient was the first from their ZIP code to be tested (within a two-week period), this feature was considered missing.

##### *Drugs*

Medication exposure, regardless of drug strength, was binarized as present or absent if recorded within the month prior to the first SARS-CoV-2 test. Drugs not observed in record were assumed as not exposed.

##### *Demographics*

Demographic variables were converted into binary features (e.g., “female” sex and “Hispanic” ethnicity), categorical features (e.g., occupation category, occupation status, county, ZIP, race, test indication), or continuous features (e.g., patient age, height, weight). We categorized patient occupation information into common categories such as “student”, “self-employed”, “unemployed”, “retired”, and “disabled” as well as the 2018 Census Occupation Code groups^2^. The shortlist of occupation codes we used was: 0010-0440, 0500-0960, 1005-1240, 1305-1560, 1600-1980, 2001-2060, 2100-2180, 2205-2555, 2600-2970, 3000-3550, 3601-3655, 3700-3960, 4000-4160, 4200-4255, 4330-4655, 4700-4965, 5000-5940, 6005-6950, 6200-6950, 7000-7640, 7700-8990, 9005-9430. Rare occupation categories or statuses (appearing in <1% of patients) were mapped to “other”.

##### *Insurance*

The insurance status was based on the insurance present at the time of the test. Patients’ payment method (Insurance) at the time of their first SARS-CoV-2 test was categorized as either “Medicaid”, “Medicare”, “Military”, “Commercial”, “Self-pay”, and/or “other”, or “null” binary features. This was not treated as a categorical feature as a patient could have multiple insurance types present.

##### *Test indication*

Testing indications were categorized into five binary features. At the time the SARS-CoV-2 RT-PCR test was ordered, providers can enter an indication for the test. Patients presenting symptoms described by CDC guidelines would be labeled 1) “Symptomatic testing.” If otherwise asymptomatic patients were admitted for care or medical procedures, they may be screened for 2) “Admission surveillance” or 3) “Preprocedural screening,” respectively. For all other reasons, asymptomatic patients that request a test may be labeled 4) “Other asymptomatic screening.” Tests where no test indication was provided were labelled as 5) “Other” or “Unknown”.

## Imputation and feature-level filtering

Where needed, we performed mean or mode imputation for continuous and categorical variables respectively. Imputation statistics were computed on the training set. We removed highly collinear variables (correlation coefficient > 0.8) after imputation. For the gradient-boosted tree, which doesn’t require imputation, we used imputation to identify collinear variables, but did not use the imputed data to train or evaluate the model. In all cases, we excluded data recorded after the first SARS-CoV-2 test to avoid information leakage.

### Modelling details

We randomly split the data into three datasets: training data (*n* = 40943), validation data (*n* = 12847), and evaluation data (*n* = 10206). The evaluation data was not accessed until the end of the study and was used only to report model performance. The training and validation data were used for model fitting, evaluation during model development, and hyperparameter selection including early stopping point.

We trained a logistic regression model as a baseline comparison model – this is shown in Fig A1. The data given to the logistic regression model was imputed where missing but was otherwise identical to what the gradient-boosted tree used. We see that while the ROC curves are similar, the gradient-boosted tree typically outperforms logistic regression, with a more pronounced effect in the precision-recall curve.


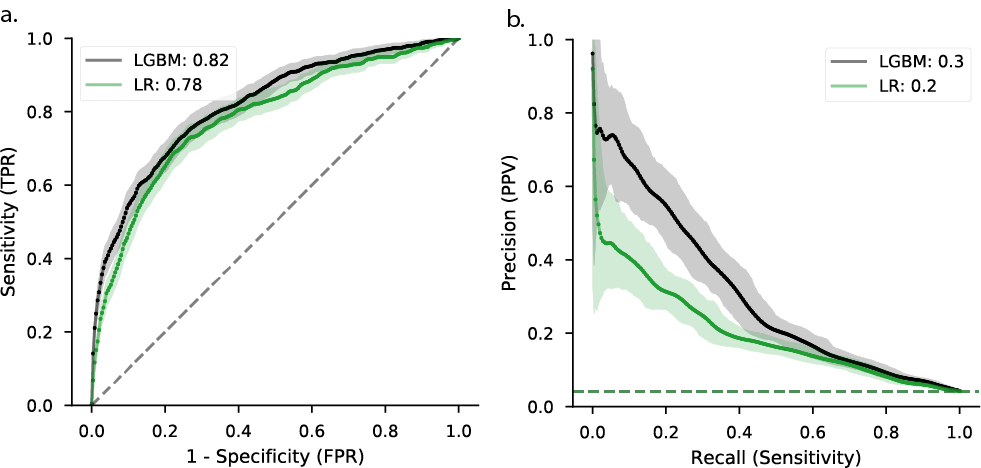


**Fig A1. Comparison of performance between gradient-boosted tree model and logistic regression.**

(A) Receiver-operating characteristic (ROC) curve. (B) Precision-recall curve. LGBM = Gradient-boosted tree model, LR = logistic regression. Solid curves show mean values from 1000 bootstrap replicates on the held-out evaluation set, and shaded regions show 95% confidence intervals.

We tested a range of hyperparameters for the gradient-boosted tree model. Model selection was performed using best AUROC on the validation set. The values tested and final choices are shown below:

| Hyperparameter | Range of values tried | Final value |
| --- | --- | --- |
| Learning rate | 0.001, 0.01, 0.1, 1.0 | 0.1 |
| Number of leaves | 5, 10, 50, 100, 200, 500, 1000 | 10 |
| Minimum data in leaf | 10, 50, 100, 200, 500, 1000, 10000 | 1000 |

We performed up to 10,000 training iterations, and used validation set performance to select an early-stopping point to avoid overfitting. To account for imbalance between positive and negative cases, we up-weighted positive examples during training (class_weight = “balanced” setting in LightGBM).

## Implementation details

All modeling and analyses were performed within an Azure Digital Research Environment and within compliance of HIPAA privacy protection regulations. Analysis code was written in Python 3.8. We made extensive use of scientific Python libraries, including NumPy v1.19.2,^3^ Pandas v1.0.3,^4^ and Matplotlib v3.1.3.^5^ Model training and evaluation made use of LightGBM v3.0.0^6^ and Sci-kit learn v0.23.1.^7^

Owing to the high dimensionality of the resulting model, sharing the full model weights poses a privacy risk as individual trees may identify patients from unique combinations of features. The methodology described in this paper could be followed and a model trained on locally-available data. This will mitigate both privacy risks and dataset shift between geographic locales.

## Individual feature importance stratified by visit type

In Fig A2, we reported SHAP values across all patients in the evaluation set and reported aggregated feature importance by visit type. In this section, we report the feature-level SHAP value distributions stratified by the three visit types. This is possible because SHAP provides *example*-level explanations, so we can inspect the distribution of SHAP values for any subset of examples.


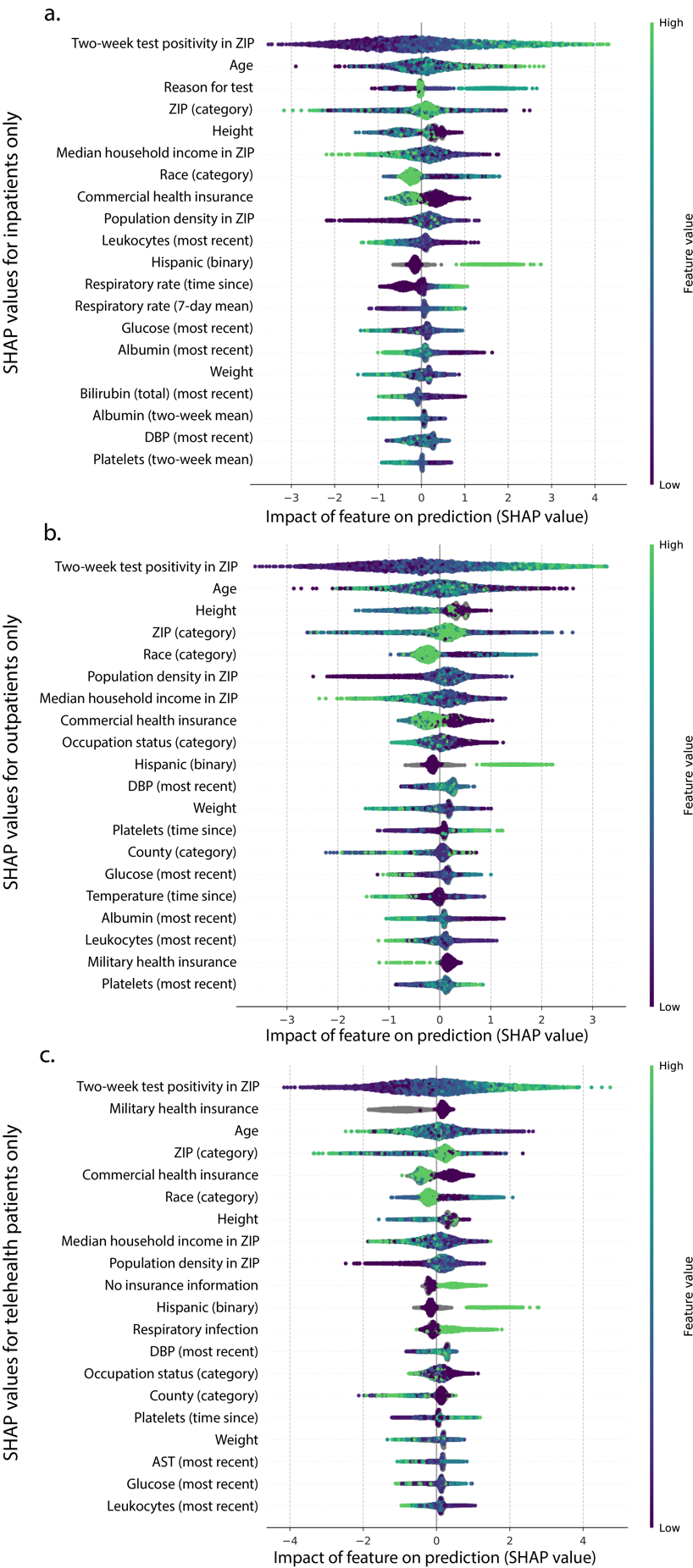


**Fig A2. SHAP values for patients stratified by visit type**.

Top to bottom (panels a-c): inpatients, outpatients, telehealth patients. DBP = diastolic blood pressure; AST = alanine aminotransferase.

Table A1 below provides the list of features used in the model, including their categorization, missingness, and indication of inclusion in “minimal” and “intermediate” feature sets.

| feature name | category | missingness (%) | minimal set | intermediate set |
| --- | --- | --- | --- | --- |
| alcohol abuse | Chronic conditions | 0.0 |  | ✓ |
| anemia | Chronic conditions | 0.0 |  | ✓ |
| anxiety | Chronic conditions | 0.0 |  | ✓ |
| asthma | Chronic conditions | 0.0 |  | ✓ |
| atherosclerosis | Chronic conditions | 0.0 |  | ✓ |
| atrial fibrillation | Chronic conditions | 0.0 |  | ✓ |
| cardiac arrhythmia | Chronic conditions | 0.0 |  | ✓ |
| congestive heart failure | Chronic conditions | 0.0 |  | ✓ |
| copd | Chronic conditions | 0.0 |  | ✓ |
| depression | Chronic conditions | 0.0 |  | ✓ |
| diabetes | Chronic conditions | 0.0 |  | ✓ |
| gastroesophageal reflux | Chronic conditions | 0.0 |  | ✓ |
| hyperlipidemia | Chronic conditions | 0.0 |  | ✓ |
| hypertension | Chronic conditions | 0.0 |  | ✓ |
| hypokalemia | Chronic conditions | 0.0 |  | ✓ |
| hypotension | Chronic conditions | 0.0 |  | ✓ |
| hypothyroidism | Chronic conditions | 0.0 |  | ✓ |
| kidney disease | Chronic conditions | 0.0 |  | ✓ |
| obesity | Chronic conditions | 0.0 |  | ✓ |
| prediabetes | Chronic conditions | 0.0 |  | ✓ |
| renal disease | Chronic conditions | 0.0 |  | ✓ |
| vitamin D deficiency | Chronic conditions | 0.0 |  | ✓ |
| county | Geography | 0.0 | ✓ | ✓ |
| acetaminophen | Drugs | 0.0 |  | ✓ |
| acetaminophen 325 mg / hydrocodone bitartrate 5 mg oral tablet | Drugs | 0.0 |  | ✓ |
| albuterol | Drugs | 0.0 |  | ✓ |
| amlodipine | Drugs | 0.0 |  | ✓ |
| amoxicillin | Drugs | 0.0 |  | ✓ |
| aspirin | Drugs | 0.0 |  | ✓ |
| atorvastatin | Drugs | 0.0 |  | ✓ |
| azithromycin | Drugs | 0.0 |  | ✓ |
| cefazolin | Drugs | 0.0 |  | ✓ |
| ceftriaxone | Drugs | 0.0 |  | ✓ |
| cephalexin | Drugs | 0.0 |  | ✓ |
| cholecalciferol | Drugs | 0.0 |  | ✓ |
| ciprofloxacin | Drugs | 0.0 |  | ✓ |
| dexamethasone | Drugs | 0.0 |  | ✓ |
| diphenhydramine | Drugs | 0.0 |  | ✓ |
| docusate | Drugs | 0.0 |  | ✓ |
| doxycycline | Drugs | 0.0 |  | ✓ |
| enoxaparin | Drugs | 0.0 |  | ✓ |
| fentanyl | Drugs | 0.0 |  | ✓ |
| fluticasone | Drugs | 0.0 |  | ✓ |
| furosemide | Drugs | 0.0 |  | ✓ |
| gabapentin | Drugs | 0.0 |  | ✓ |
| heparin | Drugs | 0.0 |  | ✓ |
| hydromorphone | Drugs | 0.0 |  | ✓ |
| ibuprofen | Drugs | 0.0 |  | ✓ |
| insulin lispro | Drugs | 0.0 |  | ✓ |
| ketorolac | Drugs | 0.0 |  | ✓ |
| levofloxacin | Drugs | 0.0 |  | ✓ |
| lidocaine | Drugs | 0.0 |  | ✓ |
| lisinopril | Drugs | 0.0 |  | ✓ |
| lorazepam | Drugs | 0.0 |  | ✓ |
| magnesium oxide | Drugs | 0.0 |  | ✓ |
| magnesium sulfate | Drugs | 0.0 |  | ✓ |
| metoprolol | Drugs | 0.0 |  | ✓ |
| midazolam | Drugs | 0.0 |  | ✓ |
| morphine | Drugs | 0.0 |  | ✓ |
| omeprazole | Drugs | 0.0 |  | ✓ |
| ondansetron | Drugs | 0.0 |  | ✓ |
| oxycodone | Drugs | 0.0 |  | ✓ |
| pantoprazole | Drugs | 0.0 |  | ✓ |
| polyethylene glycol 3350 | Drugs | 0.0 |  | ✓ |
| polyethylene glycol 3350 236000 mg / potassium chloride 2970 mg / sodium bicarbonate 6740 mg / sodium chloride 5860 mg / sodium sulfate 22740 mg powder for oral solution | Drugs | 0.0 |  | ✓ |
| potassium chloride | Drugs | 0.0 |  | ✓ |
| prednisone | Drugs | 0.0 |  | ✓ |
| sennosides | Drugs | 0.0 |  | ✓ |
| sodium bicarbonate | Drugs | 0.0 |  | ✓ |
| sodium chloride | Drugs | 0.0 |  | ✓ |
| sulfamethoxazole 800 mg / trimethoprim 160 mg oral tablet | Drugs | 0.0 |  | ✓ |
| triamcinolone | Drugs | 0.0 |  | ✓ |
| vancomycin | Drugs | 0.0 |  | ✓ |
| female | Demographics | 0.0 | ✓ | ✓ |
| hispanic | Demographics | 16.8 | ✓ | ✓ |
| insurance: commercial | Insurance | 16.9 |  | ✓ |
| insurance: medicaid | Insurance | 16.9 |  | ✓ |
| insurance: medicare | Insurance | 16.9 |  | ✓ |
| insurance: military | Insurance | 16.9 |  | ✓ |
| insurance: null | Insurance | 16.9 |  | ✓ |
| insurance: other | Insurance | 16.9 |  | ✓ |
| insurance: selfpay | Insurance | 16.9 |  | ✓ |
| albumin (most recent) | Labs | 52.4 |  |  |
| albumin (14D mean) | Labs | 82.6 |  |  |
| ALT (most recent) | Labs | 50.4 |  |  |
| ALT (14D mean) | Labs | 81.6 |  |  |
| anion gap (most recent) | Labs | 43.1 |  |  |
| anion gap (14D mean) | Labs | 75.3 |  |  |
| aPTT (most recent) | Labs | 75.1 |  |  |
| aPTT (14D mean) | Labs | 89.9 |  |  |
| AST (most recent) | Labs | 50.5 |  |  |
| AST (14D mean) | Labs | 81.7 |  |  |
| bilirubin (total) (most recent) | Labs | 50.3 |  |  |
| bilirubin (total) (time since) | Labs | 50.3 |  |  |
| chloride (most recent) | Labs | 42.8 |  |  |
| chloride (14D mean) | Labs | 75.1 |  |  |
| cholesterol (most recent) | Labs | 89.3 |  |  |
| creatinine (most recent) | Labs | 42.7 |  |  |
| CRP (most recent) | Labs | 85.9 |  |  |
| CRP (time since) | Labs | 85.0 |  |  |
| eGFR (most recent) | Labs | 48.8 |  |  |
| eGFR (14D mean) | Labs | 86.8 |  |  |
| erythrocyte (most recent) | Labs | 42.6 |  |  |
| erythrocyte (14D mean) | Labs | 74.1 |  |  |
| ferritin (most recent) | Labs | 85.7 |  |  |
| ferritin (time since) | Labs | 85.7 |  |  |
| glucose (most recent) | Labs | 43.2 |  |  |
| glucose (14D mean) | Labs | 75.4 |  |  |
| hemoglobin (most recent) | Labs | 42.5 |  |  |
| hemoglobin (14D mean) | Labs | 74.0 |  |  |
| leukocytes (most recent) | Labs | 42.7 |  |  |
| lymphocytes (most recent) | Labs | 54.4 |  |  |
| lymphocytes (14D mean) | Labs | 81.4 |  |  |
| neutrophils (most recent) | Labs | 54.4 |  |  |
| neutrophils (time since) | Labs | 54.4 |  |  |
| neutrophils (14D mean) | Labs | 81.4 |  |  |
| platelets (most recent) | Labs | 42.6 |  |  |
| platelets (time since) | Labs | 42.5 |  |  |
| platelets (14D mean) | Labs | 74.0 |  |  |
| potassium (most recent) | Labs | 42.8 |  |  |
| potassium (14D mean) | Labs | 75.1 |  |  |
| prothrombin time (most recent) | Labs | 71.1 |  |  |
| prothrombin time (time since) | Labs | 71.1 |  |  |
| prothrombin time (14D mean) | Labs | 87.7 |  |  |
| sodium (most recent) | Labs | 42.8 |  |  |
| sodium (14D mean) | Labs | 75.1 |  |  |
| triglyceride (most recent) | Labs | 71.2 |  |  |
| triglyceride (time since) | Labs | 70.6 |  |  |
| troponin-I (most recent) | Labs | 83.5 |  |  |
| troponin-I (time since) | Labs | 83.5 |  |  |
| urea nitrogen (most recent) | Labs | 42.9 |  |  |
| urea nitrogen (time since) | Labs | 42.8 |  |  |
| median household income | Geography | 4.0 | ✓ | ✓ |
| occupation category | Demographics | 0.0 | ✓ | ✓ |
| occupation status | Demographics | 0.0 | ✓ | ✓ |
| population density in ZIP | Geography | 4.0 | ✓ | ✓ |
| race | Demographics | 0.0 | ✓ | ✓ |
| reason for test | None | 0.0 |  |  |
| age | Demographics | 0.0 | ✓ | ✓ |
| height (most recent) | Vitals | 62.9 |  | ✓ |
| weight (most recent) | Vitals | 62.0 |  | ✓ |
| abdominal pain | Symptoms | 0.0 | ✓ | ✓ |
| chest pain | Symptoms | 0.0 | ✓ | ✓ |
| constipation | Symptoms | 0.0 | ✓ | ✓ |
| cough | Symptoms | 0.0 | ✓ | ✓ |
| dehydration | Symptoms | 0.0 | ✓ | ✓ |
| diarrhea | Symptoms | 0.0 | ✓ | ✓ |
| dizziness | Symptoms | 0.0 | ✓ | ✓ |
| dyspnea | Symptoms | 0.0 | ✓ | ✓ |
| fatigue | Symptoms | 0.0 | ✓ | ✓ |
| fever | Symptoms | 0.0 | ✓ | ✓ |
| headache | Symptoms | 0.0 | ✓ | ✓ |
| hypoxemia | Symptoms | 0.0 | ✓ | ✓ |
| insomnia | Symptoms | 0.0 | ✓ | ✓ |
| migraine | Symptoms | 0.0 | ✓ | ✓ |
| muscle pain | Symptoms | 0.0 | ✓ | ✓ |
| nausea and vomiting | Symptoms | 0.0 | ✓ | ✓ |
| neck pain | Symptoms | 0.0 | ✓ | ✓ |
| pneumonia | Symptoms | 0.0 | ✓ | ✓ |
| respiratory infection | Symptoms | 0.0 | ✓ | ✓ |
| visual disturbances | Symptoms | 0.0 | ✓ | ✓ |
| visit type | All | 0.0 | ✓ | ✓ |
| DBP (most recent) | Vitals | 47.3 |  | ✓ |
| DBP (time since) | Vitals | 47.3 |  | ✓ |
| DBP (7D mean) | Vitals | 78.6 |  | ✓ |
| heart rate (most recent) | Vitals | 47.2 |  | ✓ |
| heart rate (time since) | Vitals | 47.2 |  | ✓ |
| heart rate (7D mean) | Vitals | 78.6 |  | ✓ |
| O2 sat (peripheral) (most recent) | Vitals | 48.2 |  | ✓ |
| O2 sat (peripheral) (time since) | Vitals | 48.2 |  | ✓ |
| O2 sat (peripheral) (7D mean) | Vitals | 78.8 |  | ✓ |
| respiratory rate (most recent) | Vitals | 48.4 |  | ✓ |
| respiratory rate (time since) | Vitals | 48.2 |  | ✓ |
| respiratory rate (7D mean) | Vitals | 79.2 |  | ✓ |
| SBP (most recent) | Vitals | 47.3 |  | ✓ |
| SBP (time since) | Vitals | 47.3 |  | ✓ |
| SBP (7D mean) | Vitals | 78.6 |  | ✓ |
| temperature (most recent) | Vitals | 50.0 | ✓ | ✓ |
| temperature (time since) | Vitals | 49.9 |  | ✓ |
| temperature (7D mean) | Vitals | 79.1 |  | ✓ |
| ZIP | Geography | 0.0 | ✓ | ✓ |
| 2-week test positivity in ZIP | Geography | 0.1 | ✓ | ✓ |

## **Table A1. Table of features.** List of features used in the model, indication of feature category, missingness percentage, and inclusion in “minimal” and “intermediate” feature sets. Features in the chronic conditions, symptoms, and drugs categories are not missing by construction. ALT = alanine transaminase; aPTT = activated partial thromboplastin time; AST = aspartate aminotransferase; CRP = C-reactive protein; eGFR = estimated glomerular filtration rate; DBP = diastolic blood pressure; SBP = systolic blood pressure.

These features were generated from OMOP standard concept mappings. For references to the standardized concepts and relationships, please refer to https://www.ohdsi.org/analytic-tools/athena-standardized-vocabularies/

Table A2 provides thresholds used for data quality control. Values outside these ranges were treated as erroneous and removed. Thresholds were selected loosely to remove only excessive values.

| Feature | Minimum value | Maximum value |
| --- | --- | --- |
| Age | 0 | 110 |
| Albumin | 3 | 5 |
| ALT | 0 | 250 |
| Anion gap | 0 | 20 |
| aPTT | 0 | 50 |
| AST | 0 | 250 |
| Bilirubin | 0 | 20 |
| Chloride | 0 | 300 |
| CRP | 0 | 150 |
| Glucose | 0 | 350 |
| Heart rate | 25 | 200 |
| Leukocytes | 0 | 50 |
| Lymphocytes | 0 | 50 |
| Neutrophils | 0 | 50 |
| O2 saturation | 60 | 100 |
| Platelets | 0 | 1000 |
| Prothrombin time | 0 | 5 |
| Respiratory rate | 10 | 40 |
| Temperature | 35 | 45 |
| Triglyceride | 0 | 400 |
| Urea nitrogen | 0 | 100 |
| Weight | 130 | 210 |

**Table A2. Minimum and maximum thresholds set for features.**
All variations of a feature (e.g. most recent heart rate, or average heart rate in the last week) used the same threshold.

| **Test** | **Mean (stdev) time in days between last measurement, if measured, and SARS-CoV-2 test** |
| --- | --- |
| CRP | 7.8 (8.0) |
| Bilirubin | 4.0 (6.1) |
| Ferritin | 8.2 (7.7) |
| Neutrophils | 4.3 (6.5) |
| Platelets | 3.6 (6.0) |
| Prothrombin time | 4.9 (7.1) |
| Triglyceride | 7.7 (7.2) |
| Troponin-I | 5.3 (7.2) |
| Urea nitrogen | 3.3 (5.6) |

**Table A3. Average delay between most recent lab measurements and date of SARS-CoV-2 test.**

## References

1. Voss EA, Makadia R, Matcho A, et al. Feasibility and utility of applications of the common data model to multiple, disparate observational health databases. *Journal of the American Medical Informatics Association*. 2015;22(3):553-564. doi:10.1093/jamia/ocu023

2. *2018 Census Occupation Code List with Crosswalk*. US Census Bureau; 2019. Accessed August 3, 2020. https://www2.census.gov/programs-surveys/demo/guidance/industry-occupation/2018-occupation-code-list-and-crosswalk.xlsx

3. Harris CR, Millman KJ, van der Walt SJ, et al. Array programming with NumPy. *Nature*. 2020;585(7825):357-362. doi:10.1038/s41586-020-2649-2

4. McKinney W. Data Structures for Statistical Computing in Python. In: Vol 445. Proceedings of the 9th Python in Science Conference; 2010:56-61. doi:10.25080/Majora-92bf1922-00a

5. Hunter JD. Matplotlib: A 2D Graphics Environment. *IEEE*. 2007;9(3):90-95. doi:10.1109/MCSE.2007.55

6. Ke G, Meng Q, Finley T, et al. LightGBM: A Highly Efficient Gradient Boosting Decision Tree. *Advances in Neural Information Processing Systems*. 2017;30:3146-3154. https://papers.nips.cc/paper/2017/file/6449f44a102fde848669bdd9eb6b76fa-Paper.pdf

7. Pedregosa F, Varoquaux G, Gramfort A, et al. Scikit-learn: Machine Learning in Python. *Journal of Machine Learning Research*. 2011;12(85):2825-2830. http://jmlr.org/papers/v12/pedregosa11a.html
